# Supplementary figures and images for: TDP-43 pathology in Drosophila induces glial-cell type specific toxicity that can be ameliorated by knock-down of SF2/SRSF1
Source: PLoS Genet. 2023 Sep 25;19(9):e1010973. doi: 10.1371/journal.pgen.1010973 (PMC10553832; doi:10.1371/journal.pgen.1010973)

A

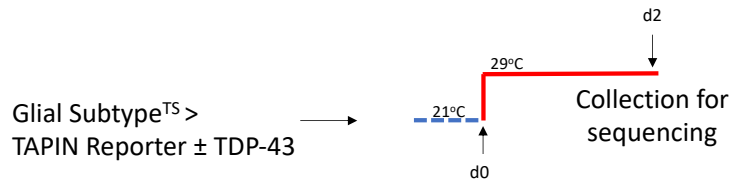

B

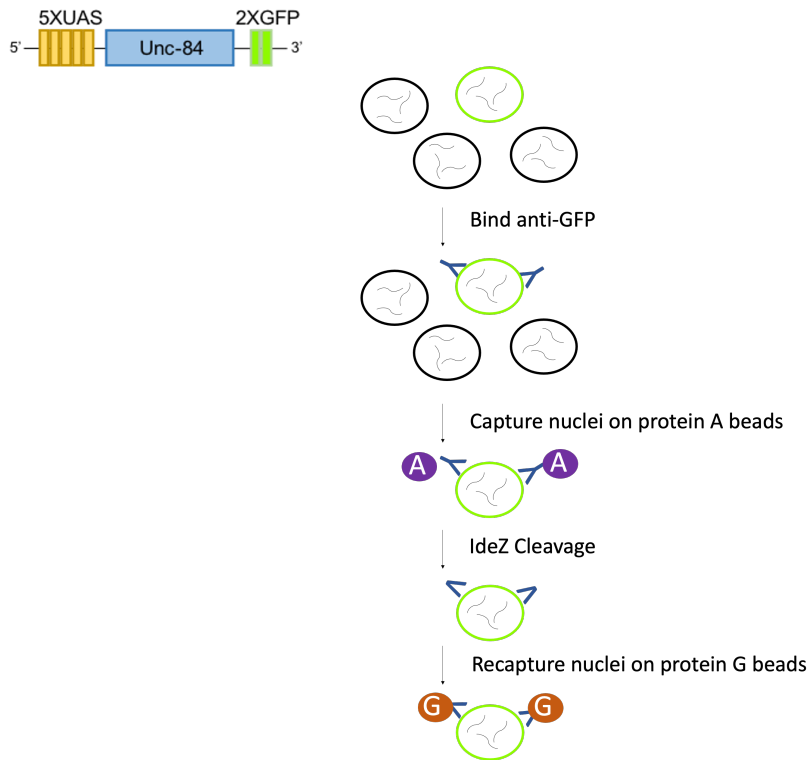

Supplement: S1 Fig — Flies expressing UAS-TDP-43 and the INTACT reporter (5XUAS-unc84-2XGFP) under control of a temperatures sensitive Gal80 plus a glial cell-type-specific Gal4 (methods) were (A) reared at 21°C and shifted to 29°C upon eclosion (day 0). Heads were collected for sequencing on day 2 and stored at -80°C until use. (B) schematic of the INTACT nuclear tag used to purify nuclei and schematic of the tandem affinity purification process. (PDF) [file pgen.1010973.s001.pdf]

A

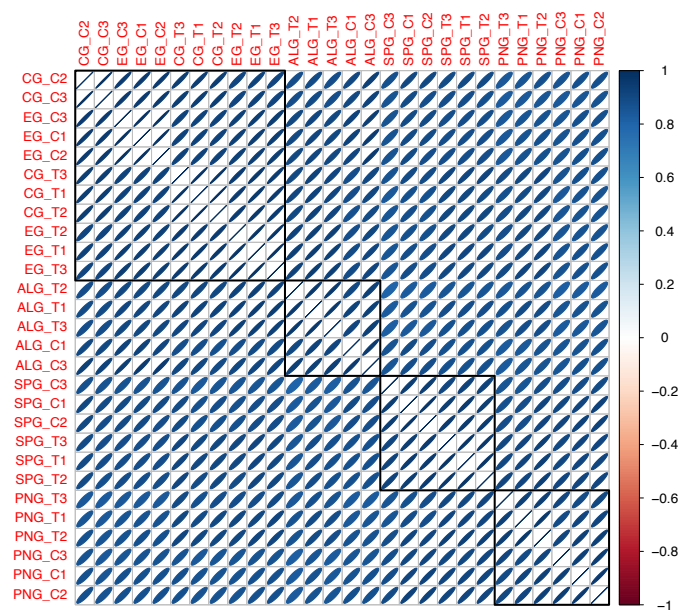

B

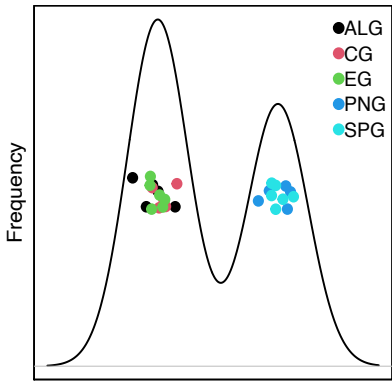

C

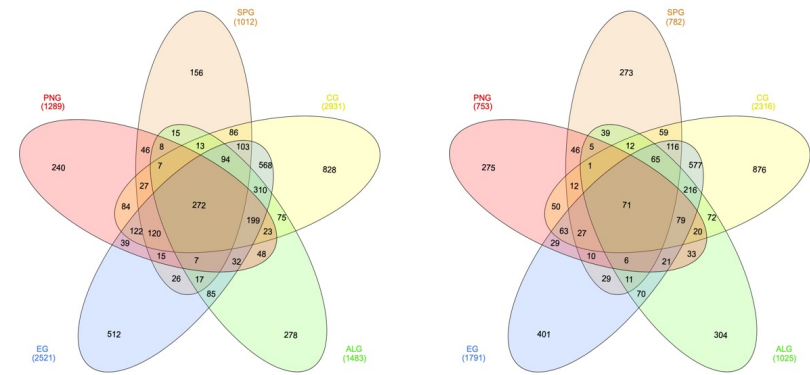

Supplement: S2 Fig — Gene expression profiles generated from (A) Pearson correlation analysis demonstrating EG and CG more closely resemble each other at baseline than they do their own cell type with TDP-43 expression (B) principle component analysis plot demonstrating PNG and SPG separate from other glial cell types. (C) upregulated and downregulated genes (p < .05) in a given glial cell type and their overlap with other glial cell types. (PDF) [file pgen.1010973.s002.pdf]

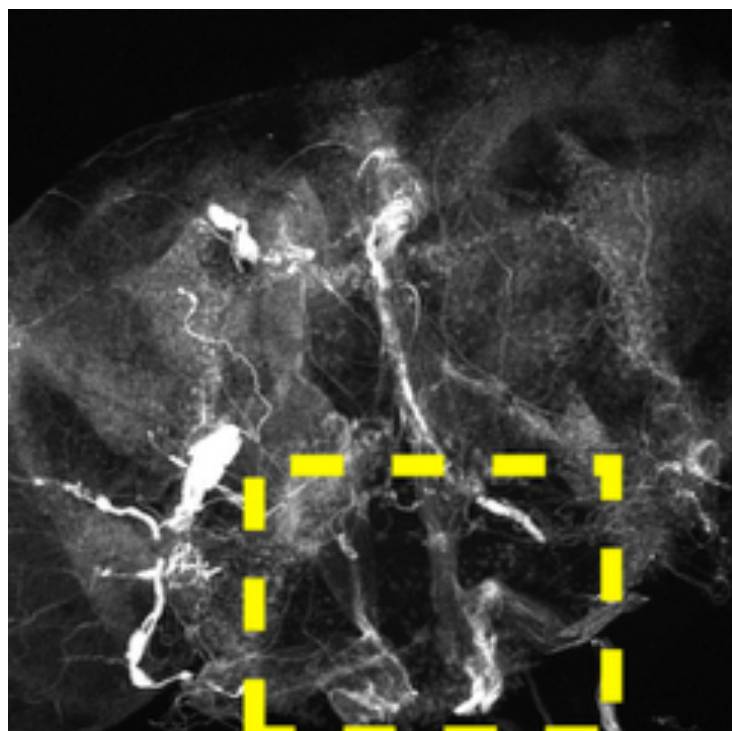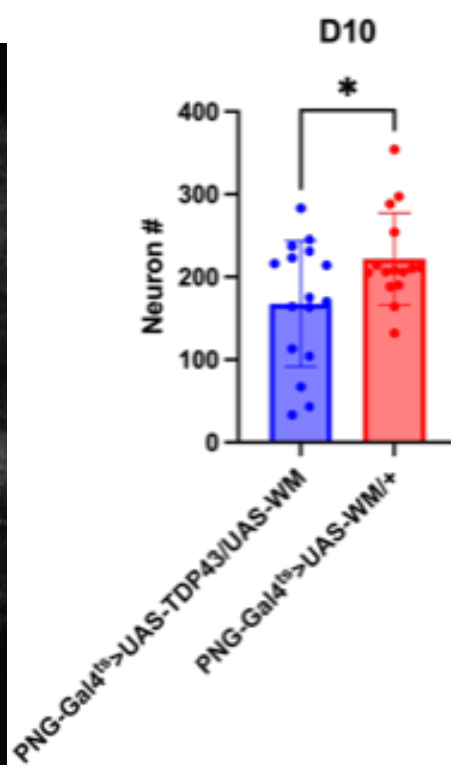

Supplement: S3 Fig — The PNG-Gal4 and Gal80ts were used to induce post development expression of hTDP-43 and UAS- Watermelon (WM), a dual reporter module that encodes both a myristylated GFP that tethers to the membrane (myr-GFP-V5) and nuclear localized mCherry (H2B-mCherry-HA). As in other figures, flies were reared at 21°C and shifted to 29°C upon eclosion (day 0). To quantify effects on neuron number, brains were dissected at day 10 (D10) post temperature shift. PNG glia were visualized using the mCherry nuclear reporter, and neuronal nulei were visualized using the Elav antibody. The numbers of Elav-labeled neuronal nuclei were quantified in A 4.68 μm stack (10 sections, 0.52 μm intervals) of ventral, posterior brain region as depicted with a yellow box in the image at left. This reveals a statistically significant (N = 15; Student’s T-test; P = 0.0319) reduction in numbers of neurons in TDP-43 expressing (PNG-Gal4ts:UAS-TDP-43/UAS-WM) vs controls (PNG-Gal4ts>UAS-WM). Means and SEM are shown. (PDF) [file pgen.1010973.s003.pdf]
