# Supplementary material for: TDP-43 pathology in Drosophila induces glial-cell type specific toxicity that can be ameliorated by knock-down of SF2/SRSF1
Source: PLoS Genet. 2023 Sep 25;19(9):e1010973. doi: 10.1371/journal.pgen.1010973 (PMC10553832; doi:10.1371/journal.pgen.1010973)
Supplement: S1 Table — (PDF) [file pgen.1010973.s004.pdf]

Glial Subtype	Cells/ Brain[1]	Cells/ Group (n=6)	Flies/ Group (n=6)
PNG	2246	>1,000,000	>445
SPG	300	>300,000	>1,000
CG	2635	>1,000,000	>380
ALG	4618	>1,000,000	>217
EG	3722	>1,000,000	>267

S1 Table. Numbers of fly heads used for TAPIN purification and sequencing based on number of glial cells of each type per brain.[1]Kremer et al., 2017
