# Supplementary material for: TDP-43 pathology in Drosophila induces glial-cell type specific toxicity that can be ameliorated by knock-down of SF2/SRSF1
Source: PLoS Genet. 2023 Sep 25;19(9):e1010973. doi: 10.1371/journal.pgen.1010973 (PMC10553832; doi:10.1371/journal.pgen.1010973)
Supplement: S8 Table — (PDF) [file pgen.1010973.s011.pdf]

Metric	PNG^{ts} Control (n=3)	PNG^{ts} + TDP-43 (n=3)	SPG^{ts} Control (n=3)	SPG^{ts} + TDP-43 (n=3)	CG^{ts} Control (n=2)	CG^{ts} + TDP-43 (n=3)	ALG^{ts} Control (n=2)	ALG^{ts} + TDP-43 (n=3)	EG^{ts} Control (n=3)	EG^{ts} + TDP-43 (n=3)
SF2 expression (MRN)	899	363	1090	594	780	727	591	453	780	669
Log2 fold change	-1.31		-0.85		n/a		n/a		n/a	
p	<0.0001		<0.001		n/a		n/a		n/a	

S8 Table. SF2 expression across glial cell types. SF2 expression was significantly reduced at day 2 post induction of TDP-43 in SPG and PNG day 2.
