# Supplementary material for: TDP-43 pathology in Drosophila induces glial-cell type specific toxicity that can be ameliorated by knock-down of SF2/SRSF1
Source: PLoS Genet. 2023 Sep 25;19(9):e1010973. doi: 10.1371/journal.pgen.1010973 (PMC10553832; doi:10.1371/journal.pgen.1010973)
Supplement: Attachment — Submitted filename: responses to reviewers.pdf [file pgen.1010973.s012.pdf]

We were pleased to receive such supportive and constructive comments from all three reviewers. Our revised manuscript has addressed each critique with significant edits to the manuscript and figures, as well as addition of new data that is now shown as Fig.S3.

Below is a point-by-point response outlining how we have responded to each each reviewer comment:

Reviewer #1: Krupp et al. described the effect of SF2/SRSF1 knockdown in glia-induced TDP-43 pathology in Drosophila. Overall the study is interesting, of interest, and well conducted. I have some major concerns prior to publication:

1. How physiologically relevant is glial-specific TDP-43 overexpression? TDP-43 accumulation occurs in both glia and neurons, but is TDP-43 expressed in both cell types? If it's dominantly expressed in neurons, overexpressing TDP-43 in glia does not recapitulate the situation under normal physiological condition.

**This is an important question. TDP-43 is normally expressed in both glial and neuronal cells, and the protein pathology is observed in both neurons and glial cells. Our introduction section includes the following statement and citations:**

**First, pathological TDP-43 in patients is observed both in neurons and in glial cells[2,29-32] and there are established roles in disease progression of both astrocytes and microglia[33-37].**

2. For all experiments in the study, does the control have similar GAL4:UAS ratio as the experimental group? As titration effect of gene expression might occur when using one GAL4 for two UAS, it will be best to compare all effects with similar number of UAS transgenes for the same GAL4.

**For all cases where we compare levels of toxicity of Gal4 driven TDP-43 to toxicity of another effector, the answer is yes. For Example in Fig.3, we compare the effects of UAS-rpr vs UAS-TDP43 on cell survival or on organismal survival. In those experiments, the animals all contained both a UAS-fluorescent reporter and either the UAS-rpr or the UAS-TDP43, along with the PNG gal4 driver and the Gal80ts. Similarly, in figure 5 where we examined the effects of SF2/SRSF1 knockdown on glial cell survival or organismal survival (lifespans), we balanced the number of UAS lines. So for example, we used a Gal4 line (astrocyte, PNG or repo) plus the Gal80ts, plus the UAS-TDP43 and then compared adding either UAS-SF2-IR or mCherry-IR. This balances the number of UAS transgenes. The only exceptions to this are for the cases where we merely tested the effects of expressing TDP-43 vs not expressing TDP-43 and quantified gene expression (Fig 2, 4) or lifespan (Fig 1).**

3. Did the authors try overexpressing SF2/SRSF1 in the presence of TDP-43 in glia? It is a bit counter-intuitive to learn that knockdown of SF2/SRSF1 ameliorate TDP-43 pathology in glia, as

RNA-sequencing results suggest that this gene is downregulated when TDP-43 is overexpressed. One would imagine that by overexpressing SF2/SRSF1 TDP-43 pathology will be rescued.

**We did not test the effects of over-expressing SF2/SRSF1. We agree that in principle this would be an interesting thing to try, although we cannot predict one way or another whether it would have any impact. We focused on down-regulation because we had previously found that knocking SF2/SRSF1 down is sufficient to suppress the toxicity of TDP43. The reviewer is correct that TDP-43 proteinopathy down regulates SF2/SRSF1 in PNG, and further knock down ameliorates the toxicity of TDP-43. We don't have a mechanistic explanation for why SF2/SRSF1 is down regulated in this cell type, but we are not particularly perplexed by the fact that further down regulation is ameliorating of the toxicity given the fact that knock down of this gene has previously been reported by us, and others, as a suppressor of TDP-43 and of C9orf72 mediated neurodegeneration. Further, we observe no down regulation of this gene in astrocytes, and also suppress the toxicity there by down regulation with the same RNAi line. We hope the reviewer is satisfied with this despite the fact that the gene's down regulated in PNG is not understood.**

4. The discussion on the non-cell-autonomous toxicity from glia to neurons is a bit hard to understand. It will help if the author can revise and add in more discussion to make things more clear.

**Thanks for pointing this out, we agree that our previous discussion of non-cell autonomous effects was not as clear as we had intended. We have significantly revised this section of the discussion and hope it is more clear.**

5. There is a typo in Figure 1A, the genotype is unc8402xGFP?

**Thanks for catching this. This refers to the nuclear envelope tag that was used to purify nuclei for nuclear RNA seq. The reviewer is correct that the nomenclature does not perfectly match what is used throughout the rest of the text. We have edited the figure so that it now reads 5XUAS-Unc84-2XGFP to be compatible with the nomenclature in the text.**

Reviewer #2: Understanding how TDP-43 related pathology and aggregation can underlie neurodegenerative diseases like ALS, FTD, and AD is a critical current goal for the field. Important recent work highlighted that not only neurons play a role in the pathological process, but that non-cell autonomous interactions with glia are important. Krupp and colleagues examine such interactions in more depth in this manuscript, to determine the effects of TDP-43 overexpression in specific subpopulations of glia on TDP-43 pathology. They show that organismal survival is most severely impacted following TDP-43 overexpression in perineurial glia or astrocytes; for perineurial glia, this is not due to cell ablation, but likely non-cell autonomous defects. Further, they observe that TDP-43 overexpression in each glial cell type

leads to varying degrees of age-dependent cell loss. The authors conduct TAPIN-seq to determine how TDP-43 overexpression affects the transcriptome of each subpopulation of glial cells and show that SF2/SRSF1 is reduced in perineurial glia and astrocytes, consistent with previous broad cell-type findings in ALS models. Further knockdown of SF2/SRSF1 in either perineurial glia or astrocytes reduces TDP-43 pathology including cell loss and lifespan.

This paper represents a strong and important contribution to the field for two main reasons: 1) it demonstrates a fascinating biological finding that glial subtypes are not equally susceptible / causative in TDP-43 pathology and 2) it establishes a valuable resource in the nuclear RNA sequencing databases of different glial subtypes under normal conditions and TDP-43 overexpression conditions. Both aspects are notable advances in the field. Moreover, the paper is well-written, with the experimental logic clearly laid out and the results straightforward and easy to follow. My criticisms are minor and largely restricted to issues of data presentation and the need for additional discussion on the roles of glial subtypes and the mechanism of SF2/SRSF1 function. With these minor additions, the paper will be suitable for publication.

Experimental Point - Though consistent with data from the field, the knockdown of SF2/SRSF1 that rescues TDP-43 pathology when TDP-43 pathology already results in a reduction of SF2/SRSF1 is counterintuitive. If the two were causative / genetically linked, I expect SF2/SRSF1 overexpression would rescue the pathology. Can you instead overexpress SF2/SRSF1 and see altered TDP-43 pathology?

**One other reviewer also made a similar point. We did not test the effects of over-expressing SF2/SRSF1. We agree that in principle this would be an interesting thing to try, although we cannot predict one way or another whether it would have any impact. We focused on down-regulation because we had previously found that knocking SF2/SRSF1 down is sufficient to suppress the toxicity of TDP43. The reviewer is correct that TDP-43 proteinopathy down regulates SF2/SRSF1 in PNG, and further knock down ameliorates the toxicity of TDP-43. We don't have a mechanistic explanation for why SF2/SRSF1 is down regulated in this cell type, but we are not particularly perplexed by the fact that further down regulation is ameliorating of the toxicity given the fact that knock down of this gene has previously been reported by us, and others, as a suppressor of TDP-43 and of C9orf72 mediated neurodegeneration. Further, we observe no down regulation of this gene in astrocytes, and also suppress the toxicity there by down regulation with the same RNAi line. We hope the reviewer is satisfied with this despite the fact that the gene's down regulated in PNG is not understood.**

Further, the authors posit that SF2/SRSF1 "rescue" results in "a reduction in the non-cell autonomous toxicity of the glia to surrounding neurons." Can this be demonstrated and quantified? It would help strengthen the existing data. It would also be helpful for the authors to include further discussion of the role / mechanism of SF2/SRSF1.

**We agree with the reviewer that our data do not directly demonstrate that the suppression of TDP-43 toxicity that we observe with SF2/SRSF1 knockdown in glia involves preventing non-cell autonomous effects on neuronal survival. To address this critique, we have made**

**two changes. First, we have added data (Fig. S3) quantifying the loss of neurons when we drive TDP-43 expression only in PNG. This demonstrates that such glial expression of TDP43 does cause loss of neurons. Second, we have moved the above statement about non-cell autonomous effects of SF2/SRSF1 knockdown from the results section into the discussion, where we make it clear that this is a hypothesis that we favor rather than a conclusion that is demonstrated by data.**

Discussion Point - There is little discussion of the role that astrocytes are playing in TDP-43 overexpression pathology. The roles of subperineurial glia and perineurial glia are well discussed, but additional discussion / speculation on the role of astrocytes would be very helpful here.

**Great point. We have added some discussion and citations to literature describing known effects of astrocyte toxicity to neurons in neurodegeneration. We do not know if the similarly toxic effects of Drosophila astrocytes in our TDP-43 model share mechanistic similarities.**

Data Presentation Points - My biggest issue with this paper is in data presentation. In its current state, it is very challenging to discern labels and interpret the results of figures and graphs throughout the paper. Though the issue is present in all figures, it is most distinctly so with Figure 2. For all figures, can the authors adjust font size and clarity? In Figure 2, even at very high magnification, it's still challenging to tell what the labels are showing. Additionally, larger graphs (i.e., 2B, D, F, H, J) would be very helpful in conveying the important points as with the rest of the figures. In all figures, larger image labels would be helpful.

**Many thanks to the reviewer for noticing this. We have increased the font sizes and plot sizes as much as was possible.**

In Figure 1, it is also difficult to tell the difference in the curve genotypes due to the size of the shapes. Can you color the TDP-43 containing curves so it's easier to see? Also, can you denote significance on the graph? There is no significance in the figure despite noting it in the text and a p value in the figure legend.

**Thanks for noticing these issues. We have made the color of the TDP-43 curves different from the controls in Fig 1, and also followed this suggestion for the lifespan curves in Fig 3 and Fig 5. As for p values, adding this to the lifespan curves is difficult because a "*" and p value would suggest a difference at a particular time-point, when in fact the log-rank statistical test used compares the curves across time points. So we worried that putting an asterisk and a p value in the figure itself could cause confusion, and instead opted to describe the stats in the legend and text. We have now made it clear in legends what test was used. We hope this is satisfactory.**

Finally, in Figure 4, the letter labeling is inconsistent with the rest of the paper. Also, the pink bar denoting the Tg_TDP43 genotype is difficult to see. Can it be changed to aid in visibility?

**Thank you for noticing these errors! These are corrected in the revised figures, and we also chose colors that we think will be easier to see.**

Reviewer #3: TDP43 pathology plays a central role in the pathogenesis of ALS and FTD, and may be also involved in AD as well. Thus, it has been under intense investigation in the field. Over the years, Dr. Josh Dubnau and his colleagues have investigated the effects of TDP43 pathology in Drosophila by overexpressing it in different cell types and have made a series of important discoveries (such as DNA damage and retrotransposon activation). In the current study, the authors made an interesting observation that TDP43 expression in the perineural glia (PNG) and astrocytes had most pronounced effect on organismal survival compared with other types of glia. Because genetic ablation of PNG did not have the same effect, these findings suggest that some unknown factors released by TDP43-expressing PNG cause systematic toxicity and neurodegeneration. This is important, because they are now in an excellent position to perform genetic studies to identify the nature of these factors released by PNG. Of course, their work here would be much more significant and novel if they could report what these factors are. Then they went on to perform cell-type specific nuclear RNA-seq and found that SF2/SRSF1 is downregulated in PNG expressing TDP-43. Further KD of SF2/SRSF1 in PNG rescued loss of these glia and reduced systematic toxicity of TDP43. Although the beneficial effects of SF2/SRSF1 KD in TDP-43 or C9 models are already known, their RNA-seq datasets will be useful for the field.

The authors **can consider** the following suggestions for minor revisions to further improve their manuscript.

1. The strength of different glial subtype-specific Gal4 drivers may be different. Is it possible different levels of TDP43 are expressed in different glial subtypes to cause different effects on organismal survival? Western blot analysis will not help address this question because the numbers and location of glia in different subtypes are different. Or immunostaining of different flies is good enough to tell the difference. At a minimal, the authors can discuss more in detail the number and location of glial subtypes, and potentially differential strengths of different Gal4 drivers.

**This is an excellent point, and we agree fully. We have added this point to the results section, where we now state:**

***"It is challenging to make direct comparisons between the severity of lethality with TDP-43 over-expression in each of the 5 glial cell types because the levels of expression may vary across cell types. But it is noteworthy that the effects on lifespan with induced TDP-43 pathology in PNG (Fig.1B) were particularly severe, and approached the magnitude of effect on lifespan seen with pan-glial hTDP-43 expression (Fig. 1G). Thus, inducing TDP-43 pathology***

***within each of the 5 glial cell types does impact survival of the animals, but the magnitude of effect on lifespan from TDP-43 pathology is most severe with PNG."***

2. As mentioned above, SF2/SRSF1 is already known to be a modifier in TDP43 and C9 models (e.g., PLoS Genet. 2021). If not too much work, the authors can include another novel modifier identified from their RNA-seq analysis to enhance the novelty of the study. Alternatively, they can perform a few more mechanistic studies to understand further how SF2/SRSF1 modifies TDP-43 toxicity. For instance, is SF2/SRSF1 required to export TDP43 mRNA from the nucleus to the cytoplasm as it does for C9 repeats as reported? Does SF2/SRSF1 overexpression enhance the phenotype? And so on.

**We appreciate these suggestions, and agree that they are important next steps, but we hope the reviewer will agree that they are beyond the scope of this publication. One of the reasons why we feel that this dataset will be impactful is that it provides the field with a rich dataset that includes both baseline expression of each glial sub-type, and also with the common and cell type specific changes in expression of each glial cell type in response to TDP-43 pathology. So we do hope that this spurs future mechanistic studies to examine roles of other DE genes, and the mechanisms by which SF2/SRSF1 impact TDP-43 pathology and/or toxicity.**

3. Maybe the authors published this in their earlier papers. In several places, the authors talk about toxicity of glial TDP43 on neurons, but they never showed any data. Is that possible to include neuron survival data at least in one figure?

**This is a good suggestion, and we now provide some additional data to substantiate the claims regarding toxicity of PNG to neurons (new figure S3). In a previous publication (Chang et al., Current Biology, 2019), we used mosaic analysis (the FRT system) to demonstrate that small clones of PNG, SPG or astrocytes that express TDP-43 were able to cause nearby neurons to die. And in a recently published paper (Chang et al., Nature Communications, 2023) we show that Gal4-driven expression of TDP-43 in SPG causes neurons to die as well. But spurred by this reviewer's suggestion, we have now quantified neuronal loss in response to post-developmental Gal4-driven TDP-43 expression in the PNG.**

4. In Figure 1 legend, it is stated that $p<0.0001$ for all panels. Is that true? This reviewer is a little bit surprised, because in Panel C, the small difference is largely due to the second data point and death occurred only few days after the experiment started. So the authors should carefully perform statistical analyses again and state in the legend what statistical methods were used. The number of flies for each panel should be stated and it is better to list p values in each panel of the figure as well.

**Thank you for noticing that our description of the statistical tests and the resulting P values was unclear. We now make it clear that the survival curves were compared using a log-rank**

**test. Indeed, the reviewer also is correct that not all the curves have the same P value. They are all less than 0.0001 as we stated, but we now state in the legends what the actual P values were for each comparison.**

5. In Figure 2 and Figure 3 legends, *p<0.5 must be an error. Is it *p<0.05? Also, please state the values are S.D. or S.E.M., what are n numbers, and by what statistical analysis in all figure legends.

**Thanks for pointing this out! Yes, 0.5 was a typo error, which has been corrected. We have now made it clear that figures 2,3,5 all show mean and S.D. And we have stated which statistical tests were used in each figure legend.**

6. In Figure 3B, the number of PNG cells at day 2 is >200, but the image does not show that many cells as in Figure 2. Please explain.

**The images in Figs 2 and 3 show representative anterior sections through the brain at approximately the same section level, and the quantification shows the number of labeled cells per anterior section. In Fig 2, the reporter used is Unc84-GFP fusion, which is tethered to the nuclear envelope. This reporter was used in Fig 2 because these were the same experiments in which we isolated nuclei for cell-type specific sequencing. By contrast, the reporter used in Fig 3 is an H2B-tagged nuclear mCherry (but shown in green). The levels of expression of these two reporters vary across cells, and are somewhat different between reporters, which may give the appearance of there being different numbers of labelled cells. But for each experiment, we counted both very bright nuclei and also nuclei that exhibit lower levels of reporter expression, and those numbers are not in conflict.**

7. In Figure 4 legend, they say “upregulated (blue) and downregulated (red) genes”. But in the figure, upregulated genes are in red and downregulated genes are in blue.

**Thank you for noticing this error! It is corrected!!**

8. Figure 5 can be reorganized to better utilize the empty spaces. Panels D and E can be new Panels A and B, and Panels A-C can be new Panels C-E.

**Thank you for this suggestion, we have re-arranged those panels to make better use of empty space in the figure.**

9. Please increase font size in all figures.

**Done.**
